# Immune Tolerance Maintained by Cooperative Interactions between T Cells and Antigen Presenting Cells Shapes a Diverse TCR Repertoire

**DOI:** 10.3389/fimmu.2015.00360

**Published:** 2015-08-07

**Authors:** Katharine Best, Benny Chain, Chris Watkins

**Affiliations:** ^1^Division of Infection and Immunity, University College London, London, UK; ^2^Centre for Mathematics, Physics and Engineering in the Life Sciences and Experimental Biology (CoMPLEX), University College London, London, UK; ^3^Department of Computer Science, Royal Holloway, University of London, London, UK

**Keywords:** immune tolerance, T cell population, dendritic cells, TCR repertoire, linear programming

## Abstract

The T cell population in an individual needs to avoid harmful activation by self peptides while maintaining the ability to respond to an unknown set of foreign peptides. This property is acquired by a combination of thymic and extra-thymic mechanisms. We extend current models for the development of self/non-self discrimination to consider the acquisition of self-tolerance as an emergent system level property of the overall T cell receptor repertoire. We propose that tolerance is established at the level of the antigen presenting cell/T cell cluster, which facilitates and integrates cooperative interactions between T cells of different specificities. The threshold for self-reactivity is therefore imposed at a population level, and not at the level of the individual T cell/antigen encounter. Mathematically, the model can be formulated as a linear programing optimization problem that can be implemented as a multiplicative update algorithm, which shows a rapid convergence to a stable state. The model constrains self-reactivity within a predefined threshold, but maintains repertoire diversity and cross reactivity which are key characteristics of human T cell immunity. We show further that the size of individual clones in the model repertoire becomes heterogeneous, and that new clones can establish themselves even when the repertoire has stabilized. Our study combines the salient features of the “danger” model of self/non-self discrimination with the concepts of quorum sensing, and extends repertoire generation models to encompass the establishment of tolerance. Furthermore, the dynamic and continuous repertoire reshaping, which underlies tolerance in this model, suggests opportunities for therapeutic intervention to achieve long-term tolerance following transplantation.

## 1 Introduction

Vertebrate immune system recognition uses antigen receptors produced by stochastic and hence unpredictable molecular recombination events. In this study, we propose a new explanation for how the T cell compartment of the immune system may use a stochastic set of receptors whose specificities are not predetermined to develop a useful repertoire. The requirements we impose are that the repertoire of antigen receptors should cover the set of non-self antigens as comprehensively as possible, in order to provide robust protection against any potential exposure to infectious pathogens. At the same time, the system must remain tolerant to the set of self-antigens and generally avoid autoimmunity. The fundamental aspect of our hypothesis is that self/non-self discrimination is an emergent property of the combined population of T cells, and cannot be linked by a one-to-one mapping to the individual binding strength spectrum of individual T cells and their receptors. The model we propose has important implications in the context of transplantation, since it suggests that the repertoire can be re-learnt throughout life, thus allowing an opportunity for long-term acquisition of graft-tolerance.

The clonal theory of immune responses, and its corollary, clonal deletion as a mechanism leading to self-tolerance, were developed primarily in the context of antibody and B cells (1). The theory was subsequently extended to T cells, and self-tolerance was proposed to result from clonal deletion in the thymus (2). Indeed thymic-tolerance induction remains a major feature of current models of T cell function. Nevertheless, a number of features of T cell recognition distinguish it from antibody recognition, and have suggested that repertoire selection may obey a modified form of rules.

A first important difference lies in the average affinity of T cell receptor (TCR) for its antigen. At least for the subset of alpha/beta receptor carrying T cells (which is the main focus of this study), which recognize major histocompatibility complex/peptide complexes (pMHC), this affinity is in the order of 10^–5^–10^–6^M, which is some three orders of magnitude less than that for antibody/antigen recognition (3). In addition, only a small proportion of the TCR-binding surface recognizes the antigenic target peptide itself, while the rest binds to the host MHC. A consequence of these characteristics is that the individual TCRs exhibit a great deal of promiscuity: many TCRs bind to the same peptide, while many peptides can be bound by the same TCR (4, 5). The combination of low individual affinities, and a large degree of cross-reactivity have led to the development of an elegant cooperative model of T cell recognition, the “quorum-sensing model” (6), which proposes that functional T cell responses are the product of cooperative interactions between T cells with different receptors. The decision of whether to respond or not is made at the population level, rather being determined solely at the level of an individual T cell/antigen presenting cell encounter.

Another fundamental distinction between T and B cells is that naive T cells require activation by antigen presented on the surface of an antigen presenting cell (APC), usually a dendritic cell. The APC provides the T cells with a high density array of MHC molecules carrying a diverse set of self and non-self peptides, but also a set of additional membrane bound and secreted signals which are necessary for productive T cell activation (7). Dendritic cells can interact simultaneously and consecutively with many different T cells (10–20 cells at any one time, and in the order of 200–400/h) forming an APC/T cell cluster (8–10). Such a cluster is an obvious candidate for the site of “quorum-sensing”, with the cluster, rather than the individual cell, acting as the unit of response. Cooperative behavior between cells within a cluster has been documented by us and others (11, 12). However, the antigen presenting activity of dendritic cells is not a static property. Dendritic cells switch from a “resting” state to an “active” state, and this transition is determined to a great extent by signals from innate immunity (13). Since resting dendritic cells do not provide the signals necessary for naive T cell activation, they become the “gate keepers” of adaptive immunity, and dendritic cell activation becomes a key decision point in determining whether an antigenic stimulus leads to immune activation. Resting dendritic cells may not only fail to induce productive T cell activation, but may actively induce tolerance (14). Indeed, subsets of immature dendritic cells have been shown to kill T cells in particular circumstances (15). The concept of tolerogenic dendritic cells underlies the influential “danger” model (16, 17), which postulates that self-tolerance results from the fact that self antigens are generally presented to T cells in the absence of innate immune responses. Thus self/non-self discrimination, at least outside the thymus, is determined as much by the dendritic cell and its interaction with innate immunity as by the T cell compartment itself.

Models for self-tolerance are still dominated by the concept of positive and negative selection operating on each individual T cell independently. The question of the mechanism for setting precise thresholds for positive or negative selection, so as to maximize response to non-self but minimize response to self, continue to be much debated (18, 19) and models have been developed that demonstrate the impact of these thresholds on the T cell response to self peptides (20, 21). The mechanisms for establishing self-tolerance outside the thymus are also debated, although “natural” T regulatory cells seem to play an important role (22, 23).

The very extensive literature on the induction of self-tolerance has generally been distinct from the smaller corpus of papers which deal specifically with repertoire generation. A number of models for repertoire generation have been proposed. The key experimental observations which all models must encompass are the persistently high diversity of the naive T cell pool (24), the ability for new clones to emerge and establish themselves in the repertoire (25), and the variable clone size which was an unexpected feature of the naive repertoire (26). The majority of previous models, which often have an “ecological” flavor, focus on clonal competition for a limited pool of presented self-antigens to drive clonal diversity and clonal size heterogeneity. Competition between T cells for access to pMHC results in stabilization of clone sizes when all available binding sites are occupied (27) and increased diversity as those T cells that are more different from others and therefore occupy a niche are favored (28, 29). In order to explain the emergence of new clones, and to prevent the development of a repertoire dominated by the clones with optimum affinities, a natural death rate of all clones is often assumed.

In the new model outlined below, we combine repertoire generation and self/non-self discrimination into a single process. We integrate cooperative behavior (quorum sensing) into the process of naive T cell repertoire generation, and explicitly model a system in which T cell receptors bind many different antigens with a range of different affinities. The model can be formalized as a linear programing (LP) optimization problem. It shows a rapid convergence to a stable state, in which self-reactivity is maintained below a fixed threshold. The model focuses on the shaping of the T cell repertoire in the absence of immune challenge, and in this work we do not consider the changes to the repertoire following activation in detail. Instead, we investigate the potential of the system to mount an immune response and introduce measures of the T cell population’s coverage of potential non-self antigens. We show that despite the restrictions imposed by the linear constraints that ensure self-tolerance, the repertoire remains diverse, coverage is preserved and the size of individual clones is heterogeneous. The diversity of the constrained repertoire becomes an important factor when challenge with foreign antigens does occur, and we find that this model is able to reshape the population to retain both TCR diversity and the potential to respond to non-self more strongly than self.

## 2 Materials and Methods

### 2.1 A simple computational model

We introduce a simple computational model, and then we consider possible variations of the model and possible underlying mechanisms.

We suppose that the T cell system “learns” in the following way to recognize self, and to react to self up to but not beyond response thresholds, which are determined by the APCs (in this study we prefer the more generic term APC, although the most important cell type in maintenance of the naive T cell repertoire is probably the dendritic cell). Each inactive APC carries a set of self-antigens bound to MHC and is continually “scanned” by T cells. Some of the TCRs of these T cells recognize one of the presented pMHC complexes on the surface of the APC; T cells scan the surface of the APC, stop for a period related to the strength of interaction with pMHC and then release themselves, allowing other cells an opportunity to assay their affinity to the presented antigens (8). In this model, we ignore any potential effects of ecological competition between T cells for pMHC binding sites in order to study the effects of the quorum sensing behavior.

We suppose that the APC can detect the strength of the antigen specific binding between each T cell and the APC, and we further hypothesize that the APC maintains a record of the total APC/T cell binding, using some (possibly leaky) integration mechanism over a sliding time window. The APC does not need to “know” which antigen has caused the T cell to bind, and still less which TCR clonotype the T cell expresses. The strength of signal in this model could arise from a combination of a strong affinity between pMHC and a specific TCR, or the presence of high concentrations of a particular pMHC. The model does not distinguish between these parameters but allocates an overall signal strength to each T cell/APC encounter.

We suppose that the APCs regulate the numbers of T cells in the following simple way. If the combined binding signal strength registered within a fixed time period by an APC exceeds some threshold value, then the APC sends a “kill signal” (either actively or passively) to each T cell that is bound currently or binds subsequently (15). These T cells, or some fraction of them, then die. Since the APC is recording the integrated signal over a sliding time window, this value will subsequently fall to below the signal threshold and the APC will then switch off the kill signal. The molecular mechanisms which could mediate such models are discussed below, but at this stage, we focus on the mathematical properties of such a model.

We implement a simplified version of the model described above. The biological validity of these assumptions and the extension of the model to more realistic but more complex scenarios are discussed later. We suppose that there are *N* different T cell clonotypes, with abundances at time *t* = 0 of $x^{0}=\left(x_{1}^{0},x_{2}^{0},\ldots ,x_{N}^{0}\right)$. In reality, the abundances would be integer counts, but in this model, we treat them as positive real numbers.

We denote the binding strength between a T cell clonotype *i* and self-peptide MHC complex (“spMHC”) *k* as *q_ik_*. We consider a model in which each (non-activated) APC presents a particular combination (or “profile”) of spMHCs. The spMHC profile *j* contains an amount *a_kj_* of spMHC *k*, and we suppose there are *M* such profiles that T cells may encounter. The overall binding strength of T cell *i* for APC profile *j* is then *b_ij_* = ∑ *_k_* *q_ik_a_kj_*. Note that when we refer to binding strength, we are describing a quantity that represents the amount of signal that the APC integrates due to the T cell-APC encounter.

Each T cell may have non-zero binding strength to many spMHC complexes, and each spMHC complex may bind to many T cells: the matrix of spMHC to T cell binding strengths *Q* = (*q_ik_*) is assumed to be sparse, non-negative, and with multiple positive entries in each row and column. The matrix of binding strengths of T cells to antigen profiles, *B* = (*b_ij_*), therefore, is non-negative, and less sparse than *Q*, because each antigenic profile contains multiple spMHC complexes. *B* is non-negative because an APC cannot present a negative amount of antigen; that is, the *a_kj_* are non-negative. Note that we do not consider the T cell to pMHC binding strengths *q_ik_* – instead we generate the T cell to APC profile binding strengths *b_ij_* by sampling from an assumed distribution, described later.

On these assumptions, the total strength with which all T cells bind to an APC with spMHC profile *j* is:

(1) $$r_{j}\left(x\right)=\sum_{i}x_{i}b_{\mathrm{ij}}$$

where **x** is the vector of clonotype abundances at time *t*. Writing **b***_j_* = (*b*_1_*_j_*, …, *b_Nj_*), we obtain:

(2) $$r_{j}\left(x\right)=b_{j}\cdot x$$

We set a threshold binding rate *τ* above which each APC will issue a kill signal to any T cell that is bound; that is, the APC presenting spMHC profile *j* issues kill signals to any T cells bound to it if *r_j_*(*x*) > *τ*. In principle, *τ* is a threshold that can be locally defined by the antigen presenting system: it can depend on the APC microenvironment or intrinsic antigen presenting parameters such as the MHC haplotypes. In this initial implementation, we have assumed *τ* is constant over all APCs. The rate at which T cell *i* is eliminated by “kill” signals from APCs of type *j* is proportional to the strength of the binding interaction of each T cell with that spMHC profile *j*, such that:

(3) Kill signals for clonotype i from APC type j =η ϕ(bj⋅x−τj)bijxi

where *η* is a rate parameter, *ϕ*(**b***_j_* ⋅ **x** − *τ_j_*) is the fraction of all T cells binding to APC *j* that receive a kill signal, and *x_i_* is the abundance of T cells of type *i*. Our hypothesis is that kill signals are only issued when the rate of binding to APCs is greater than *τ*; this hypothesis is expressed in terms of the function *ϕ*(*z*), which is some non-decreasing function such that 0 ≤ *ϕ*(*z*) ≤ 1 for all real *z*. *ϕ*(*z*) should be small or zero for *z* < 0, and we suppose that *ϕ*(*z*) rises toward 1 rapidly for *z* ≥ 0. The simplest choice for *ϕ* would be the Heaviside function *H*(*z*) = 1 if *z* ≥ 0 and *H*(*z*) = 0 if *z* < 0; a more biologically realistic function would be continuous and differentiable, such as the logistic function $\phi \left(z\right)=\frac{1}{1+\exp\left(-\alpha z\right)},$ for some suitable scale parameter α. The implementation captured in equation (3) further assumes each APC, and hence each spMHC profile *j*, occurs once, but the model is easily extended to incorporate variable APC numbers for each antigen profile.

So far, the model only has a mechanism for killing T cells: there must also be a method for T cells to multiply. Although it is clear that naive cells must see self-antigens in order to survive, the quantitative relationship between antigen-binding strength and proliferation in the context of T cell homeostatic proliferation remains unclear. Here, we adopt the simplest assumption, namely that all T cells spontaneously divide at some rate *ν*, although a model relating *ν* to binding strength could also be implemented.

Using these assumptions, we obtain that for each clonotype *i*:

(4) $${\dot{x}}_{i}=\nu x_{i}-\eta \sum_{j}\phi \left(b_{j}\cdot x-\tau_{j}\right)b_{\mathrm{ij}}x_{i}$$

so that

(5) $$\frac{{\dot{x}}_{i}}{x_{i}}=\nu -\eta \sum_{j}\phi \left(b_{j}\cdot x-\tau_{j}\right)b_{\mathrm{ij}}$$

We can demonstrate rather simply that the optimization will indeed always converge. For a suitable choice of *ϕ* the right hand side can be written as the gradient of a convex function of **x**. Observe that:

(6) $$\Phi \left(u\right)=\int_{-\infty }^{u}\phi \left(z\right)\mathrm{dz}$$

exists for plausible choices of *ϕ*, and is convex and differentiable provided that *ϕ*(*z*) is non-decreasing and continuous. Then define:

(7) $$f_{j}\left(x\right)=\Phi \left(b_{j}\cdot x-\tau_{j}\right)$$

Each *f_j_* is convex in **x**, and note that:

(8) $$\frac{\partial f_{j}\left(x\right)}{\partial x_{i}}=\phi \left(b_{j}\cdot x-\tau_{j}\right)b_{\mathrm{ij}}$$

Now define:

(9) $$F\left(x\right)=-\nu \sum_{i}x_{i}+\eta \sum_{j}f_{j}\left(x\right)$$

which is a sum of convex differentiable functions. The scalar function *F*(**x**) is constructed so that

$$\frac{\mathrm{\partial F}\left(x\right)}{\partial x_{i}}=-\nu +\eta \sum_{j}\phi \left(b_{j}\cdot x-\tau_{j}\right)b_{\mathrm{ij}}=-\frac{{\dot{x}}_{i}}{x_{i}}$$

so that the rate of change of **x** is expressed as:

$${\dot{x}}_{i}=x_{i}\frac{\mathrm{\partial F}\left(x\right)}{\partial x_{i}}$$

We can now write the rate of change of *F*(**x**) as:

(10) $$\frac{\mathrm{dF}\left(x\right)}{\mathrm{dt}}=\dot{x}\cdot \nabla F\left(x\right)$$

(11) $$=-\sum_{i}x_{i}{\left(\frac{\mathrm{\partial F}\left(x\right)}{\partial x_{i}}\right)}^{\;2}$$

(12) $$\leq 0\text{ since all }x_{i}\text{ are positive}$$

*F* is convex and differentiable, because it is the sum of convex, differentiable functions, and *F* therefore has a unique minimum in the region of interest, which is the non-negative quadrant. At this minimum, all constraints **b***_j_* **x** ≤ *τ_j_* will be approximately satisfied, provided that the growth rate *ν* is small compared to the “kill rates” from the APCs.

From equation (11), we know that the value of *F*, which includes a sum of measures of constraint violation, must decrease over time. However, it says little about the rate of convergence toward the minimum of *F*. In the Supplementary Material, we present a stronger analysis of the convergence of the process of equation (4), by identifying it with a version of the multiplicative weight updating algorithms surveyed by (30). This analysis establishes regret bounds for such updates on a possibly time-varying set of constraints. We note that equation (4) could be solved by standard differential equation methods, provided the rate of killing (and the rate of proliferation) remains constant. Under these conditions, the iterations become equivalent to a fixed time step, which can be allowed to decrease to the continuous case. However, we prefer to use the iterative algorithm we describe below because the discrete time steps are readily interpretable in terms of cellular events (e.g., T cell/APC interactions) and because the regret bounds it establishes are robust to variations in rate. The model therefore leaves open the possibility of introducing time-dependent and tissue-dependent variations in rates in future extensions of the basic model.

The implementation outlined above gives rise to a series of constraints on T cell abundances, which are captured by a series of linear inequalities as outlined above. An iterative method to solve this linear programing problem is set out below, and can be given a feasible biological interpretation. The proliferation rate *ν* is set so that in the absence of any “kill signals”, the T cell population would double in one unit of time, and the rate *η* of T cell killing is set relative to this.

1. Calculate the immune response to each profile, $r\left(j\right)\leftarrow \sum_{i}x_{i}b_{\mathrm{ij}}$ for all *j*.
2. Determine for which self-profiles the response threshold has been violated, v(j)←[r(j)>τ] for all *i*.
3. Adjust the T cell clonotype abundances, $x\left(i\right)\leftarrow x\left(i\right)\left(1+\nu \delta t-\eta \delta t\sum_{j}b_{\mathrm{ij}}v_{j}\right)$.

The multiplicative update analysis discussed in the Supplementary Material provides strong guarantees for time-varying constraints, corresponding to the case where APCs present varying combinations of antigens over time.

### 2.2 Assessing the potential for an immune response

In order to investigate the potential of the reshaped T cell population to mount an immune response to previously unencountered antigens, we create a set of new independently generated antigenic profiles, which were not part of the set on which the T cell population has been trained. We refer to these as “non-self profiles”. The binding strength of each existing TCR for each new profile is selected independently of its given affinities for all the self profiles, although the value is selected from the same probability distribution. We use these non-self profiles to test whether under our assumptions the T cell repertoire will achieve the dual objectives of maintaining self-tolerance, while at the same time maintaining as broad and strong a repertoire for non-self as possible.

Note that we do not model an immune response to these new profiles in this work. If the APC remains in a tolerogenic state, the introduction of new non-self profiles will typically violate the constraints, but this will result in additional T cell killing and the system will gradually readjust to remain within the immune activation threshold. We envisage that if the APC were switched to an immunogenic state (for example by exposure to innate immune danger signals) then crossing the threshold would result in activation of all APC bound T cells, resulting in an effector immune response.

We measure the ability of the T cell population to respond to a non-self profile as the total potential T cell response, calculated as *r_j_*(**x**) = ∑ *_i_* *x_i_c_ij_* for non-self profile *j*, where *C* = (*c_ij_*) is the matrix of binding strengths between T cell clonotypes and non-self profiles. It is important to note that we are not simulating the behavior of the T cell population on immune challenge here, but assessing the potential of the reshaped repertoire to respond to previously unencountered profiles. In order to measure the “success” of the reshaped repertoire, we can consider its coverage of the potential non-self antigen space. The first coverage measure we use in this study is the ratio of the mean total response against non-self profiles to the mean total response against self profiles: $\text{coverage}=\frac{\bar{r_{\mathrm{ns}}}}{\bar{r_{s}}}$ for self profiles *s* and non-self profiles *ns*. Alternatively, we also measure the coverage as the proportion of non-self profiles that give a potential T cell response greater than the average response to self profiles, i.e., $\left|\left\{\mathrm{ns}:r_{\mathrm{ns}}>\bar{r_{s}}\right\}\right|$ expressed as a fraction of the total number of non-self profiles modeled.

## 3 Results

### 3.1 Clone size adjustment algorithm reaches a solution of the repertoire constraints: Violations are resolved rapidly and repertoire is optimized slowly

**Figure 1:**
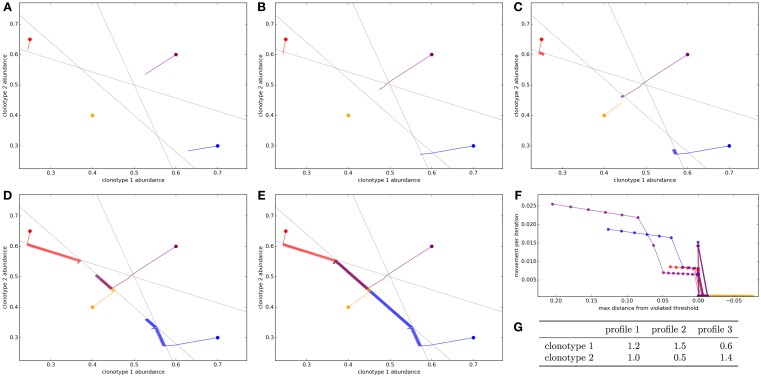
**Optimization of the T cell population to avoid autoimmunity while maximizing T cell numbers in a simplified system with two clonotypes and three spMHC profiles**. The update algorithm is initiated with different initial clonotype abundances each represented by a different color. The colored lines track the changes in clonotype abundance over iterations of the update algorithm **(A–E)**. The clone abundances after **(A)** 5, **(B)** 10, **(C)** 100, **(D)** 1,000, or **(E)** 10,000 iterations of the update algorithm. The gray lines indicate the constraint that total T cell response should be less than the threshold for each of the spMHC profiles **(F)**. For each of the starting repertoire configurations, the relationship between the Euclidean distance moved by the repertoire configuration in an iteration of the update algorithm and the distance from the furthest violated threshold, or if there are no violations the distance to the nearest threshold **(G)**. The affinities between clonotypes and spMHC profiles. Other model parameters for all panels are: *τ* = 1, the self-response threshold for each spMHC profile, *ν* = ln 2 *δt*^–1^, the growth rate and *η* = 0.01001 *δt*^–1^, the learning rate.

We first simulate a very simplified repertoire to allow us to visualize the action of the update algorithm. We start with two T cell clonotypes and three spMHC profiles. The clonotypes have binding strengths for each of the profiles as detailed in Figure 1G. In this simulation, each profile is given the same total response threshold (=1), above which there will be harmful autoimmunity. The other parameters of the update algorithm are set out in the legend of Figure 1.

The self-response thresholds for each profile and the binding strengths between clonotypes and spMHC profiles (Figure 1G) give constraints on allowable repertoires. If *x_i_* is the abundance of clonotype *i*, to avoid autoimmunity we require that:

$$\begin{array}{llll} & 1.2x_{1}+1.0x_{2}\leq 1\; & & \;\\ & 1.5x_{1}+0.5x_{2}\leq 1\; & & \;\\ & 0.6x_{1}+1.4x_{2}\leq 1\; & & \; \end{array}$$

We repeatedly simulate the update algorithm with different starting repertoire configurations. Each starting configuration is represented as a color in Figure 1. The panels in this figure show a time course of the update algorithm working on each initial repertoire configuration.

We see that if the initial repertoire configuration violates one or more of the response constraints, the update algorithm very quickly shapes the repertoire (by adjusting clonotype abundances) to a point where there is no autoimmunity (Figure 1B, 10 iterations). By contrast, when no threshold is violated by the starting configuration of the repertoire (yellow path in Figure 1), the repertoire does not move very far from the initial configuration in the first cycles.

Once the repertoire has been moved to a configuration where all constraints are satisfied, the update algorithm continues to allow each clonotype to grow as abundant as possible while remaining inside the “feasible region” (Figures 1C–E). For this arrangement of affinities, the “optimum” repertoire in terms of having the highest total abundance while avoiding autoimmunity is at a single vertex of the feasible region, and we can see that the update algorithm moves each of the initial repertoires slowly toward this point.

The speed which the clonotype abundances are adjusted is dependent on the severity of the violation of the thresholds, as the update rule is designed to do through the negative learning rate, *η*. This can be quantified by considering the Euclidean distance moved by the repertoire configuration in a timestep as a function of the Euclidean distance by which the current configuration violates a threshold (Figure 1F). There is a strong positive relationship between the severity of the violation and the speed with which the update algorithm adjusts the clonotype abundances.

### 3.2 Positive selection of clonotypes based on self-profile binding strength is required for successful immune-tolerance

**Figure 2:**
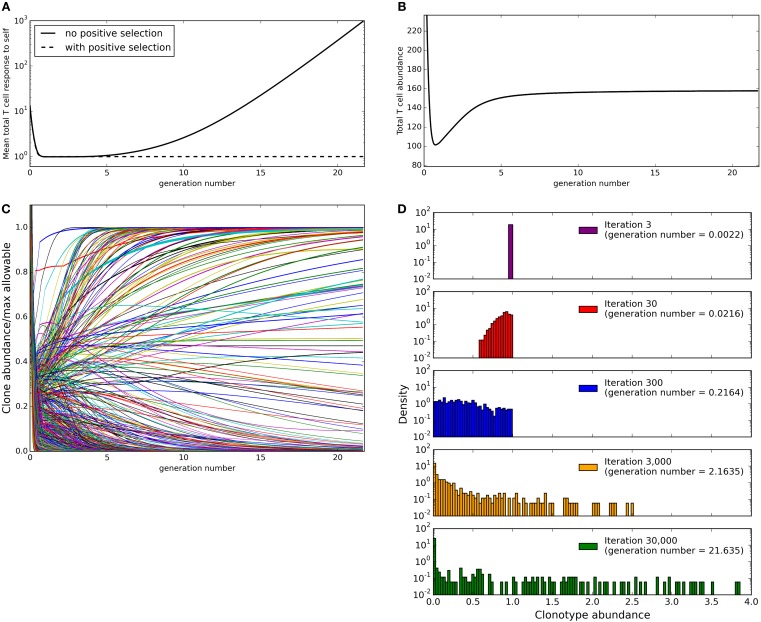
**Evolution of the repertoire under the constraints of dendritic cell dependent T cell deletion**. **(A)** mean total T cell response to spMHC profiles over time as clone sizes are updated according to the basic immune-tolerance learning algorithm with or without positive selection implemented. The total T cell response to a profile is calculated as the sum of abundance × binding strength for each clonotype. **(B)** the total T cell abundance (being the sum of the abundances of clonotypes present at a particular time) in the simulation after T cell positive selection is implemented **(C)** the abundance of each T cell clonotype over time after T cell positive selection is implemented expressed as a fraction of the maximum size the clone could reach in the absence of other clones without violating any self-response thresholds. **(D)** the clone size distribution after the indicated amount of time after T cell positive selection is implemented. Model parameters for all panels are: self-response threshold *τ* = 1, growth rate *ν* = ln 2 *δt*^−1^, learning rate *η* = 0.002001 *δt*^−1^, number of spMHC profiles *M* = 100, number of T cell clonotypes *N* = 1,000, proportion of non-zero affinities *γ* = 0.01.

We next simulated the update algorithm with a larger number of T cell clonotypes and spMHC profiles (Figure 2). For each clonotype-profile pair, the binding strength (*b_ij_* for clonotype *i* and profile *j*) is set to zero with probability 1 − *γ*. If the binding strength is not set to zero it is selected at random from a left-censored normal distribution. For simplicity, we set the response threshold to be equal to 1 for all self profiles.

We run the update algorithm and record the abundance of each clonotype at each iteration. Note that under our constant growth rate assumption, iterations can be thought of as directly equivalent to T cell generations. We set the growth rate ν such that one unit time is equal to one T cell generation, giving one T cell generation in approximately 1387 iterations. The total T cell response to a spMHC profile can be calculated as the sum of abundance × binding strength for each T cell clonotype (*r_j_* = ∑ *_i_ x_i_b_ij_* for spMHC profile *j*). We can then define successful immune tolerance as the reshaping of the T cell population into one where the total T cell response to any spMHC profile (*r_j_* for self-profile *j*) is below the threhsold *τ*. The mean total response to spMHC profiles over time (Figure 2A, solid line) is initially well controlled at the allowed threshold. However, after approximately 10 generation times algorithm, the control of the response breaks down and there is an increased average response to self, above the allowable threshold.

We noted that those clonotypes that are highly abundant after running our simulation for 30,000 cycles of the update algorithm have low maximum binding strength to spMHC profiles. We can see the reason for breakdown of control of self response if we consider a clonotype of abundance 1 that has zero binding strength for all self profiles except one, for which it has binding strength *b*. Then after one iteration of the update algorithm, the clonotype will have abundance (1 + *ν*) or (1 + *ν δt* − *η δt b*) depending on whether the total T cell response to the profile for which it has non-zero binding strength is below the allowable self-response threshold *τ* or not. In order to avoid uncontrolled growth of the clonotype, we would require that (1 + *ν δt* − *η δt b*) < 1, which is equivalent to requiring that *b* > *ν*/*η*. Therefore, we suggest that the inability of the update algorithm to control average self response is due to the presence of clonotypes for which the maximum binding strength to any of the self profiles is below *ν*/*η*. This indicates the requirement for some form of positive selection.

In its simplest form, positive selection would take the form of a function which deletes all clones whose maximum binding strength for any self profile is below *ν*/*η*. A more realistic function could make the growth rate in any one cycle depend on the average binding strength to self profiles or to the maximum binding strength to a randomly selected sample of “encountered” self profiles. In the following work, we implement the simplest form of the affinity-dependent selection, by eliminating all clonotypes with maximum binding strength to self profiles below *ν*/*η* before the update algorithm begins.

We implement this positive selection of clonotypes and re-run the simulation with the same parameters (detailed in Figure 2 legend). The total T cell response to self profiles is now tightly controlled at the allowable threshold (Figure 2A, dashed line). It is, however, possible under this model that if a clonotype escapes positive selection it slowly increases in size indefinitely.

### 3.3 Total population size homeostasis but increased clonotype abundance heterogeneity as a function of time

Naive TCR repertoires are made up of clonotypes with a broad range of abundances. We therefore examined the abundance distribution produced by the model presented in this paper. Since our implementation of the model uses continuous rather than discrete abundances, abundances never reach zero but become arbitrarily small. In order to consider the abundance distribution, we therefore set a lower threshold below which a clone is considered to be deleted. In this work, we consider a clonotype to be completely absent when its abundance falls below a threshold defined by *N*/10^8^ where *N* is the number of clonotypes in the simulation. This threshold was chosen based on consideration of a mouse immune system, which has in the order of 10^8^ T cells in total. If *N* different clonotypes of equal abundance are present in this repertoire, each clonotype could be considered to have a starting abundance of 10^8^/*N*. Hence, if a clone contracts by a factor of >10^8^/*N*, its abundance would fall below 1 and hence the clonotype can be considered as eliminated. Since the abundance of each clonotype at the start of the model is arbitrarily initiated at a value of one, this is equivalent to defining a clone with an abundance of lower than *N*/10^8^ as deleted.

We first considered the total size of the T cell compartment as a function of time. At every timepoint during the simulation, we can calculate the total size of the repertoire as the sum of the clonotype abundances that are above the “presence” threshold of *N*/10^8^ (Figure 2B). We see that this initially contracts as self-response constraint violations are resolved, but then expands (driven by the positive learning rate increasing the abundance of each clonotype when constraints are not violated) until a stable level is reached where growth and negative selection are balanced. If all other parameters of the model are fixed, the eventual total size of the T cell compartment at homeostasis is strongly correlated to the number of clonotypes present in the repertoire at the beginning of the simulation.

We then consider the abundances of individual clonotypes. The maximum allowable size for a clonotype in the model can be defined as the self-response threshold divided by the maximum binding strength that the clonotype has for any self profile, i.e.,

$$m_{i}=\frac{\tau }{\underset{j}{\max}b_{\mathrm{ij}}}$$

is the maximum allowable size for clonotype *i*. For each of the clonotypes in the simulation, we consider its abundance (expressed as a proportion of the maximum allowable abundance *m_i_* for that clonotype) across time (Figure 2C). Some clonotypes are present close to their maximum allowable size *m_i_*, presumably due to lack of cross-reactivity with other profiles or other clonotypes, while some clonotypes are quickly removed from the repertoire. It is interesting to note that while the total T cell abundance stabilizes rapidly (Figure 2B), the individual clonotype sizes remain dynamic even in later stages of the simulation. The clone size distribution (Figure 2D) spreads to include smaller clonotypes during the initial part of the simulation, then starts to include larger clonotypes as well in later iterations. At the end of our simulation, there is a large spread of clone sizes in which large and small clones co-exist, as observed experimentally, rather than a repertoire completely dominated by a few large clonotypes.

### 3.4 Increased number of T cell clonotypes provides greater repertoire coverage

**Figure 3:**
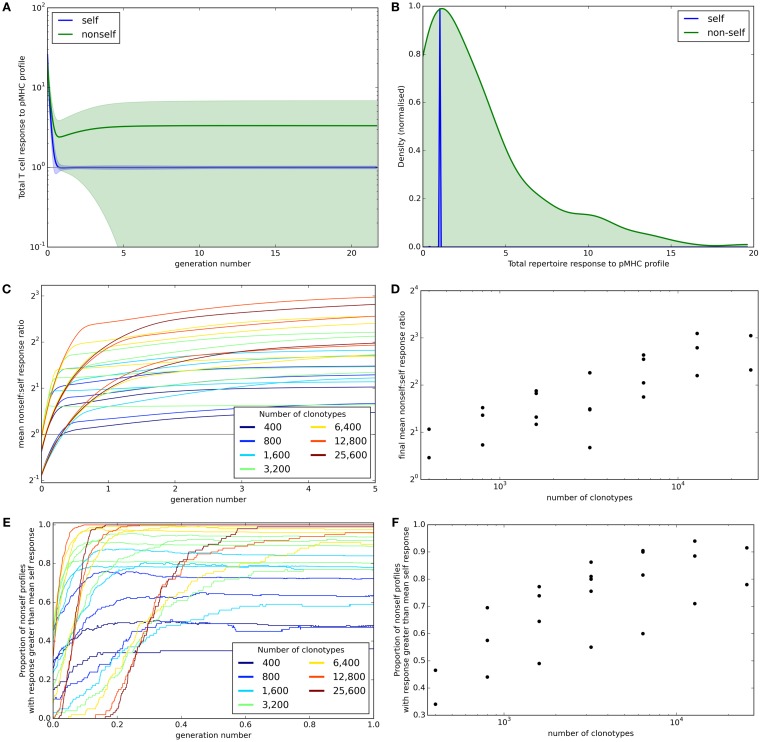
**Broad coverage to non-self is maintained during the development of a self-tolerance repertoire**. **(A)** The mean (±standard deviation) total T cell response to self (blue) or non-self (green) pMHC profiles over time with *N* = 2,000 and *M* = 200. **(B)** After 30,000 iterations of the update algorithm with parameters as in **(A)**, the distribution of total T cell response to self (blue) and non-self (green) pMHC profiles. **(C)** The ability of the repertoire to successfully mount an immune response to non-self pMHC profiles, measured as the average total response to a non-self profile divided by the average total response to a self profile, over time. The number of T cell clonotypes in a simulation is indicated by color, with the number of self profiles simulated ranging between 100 and 800. **(D)** The relationship between number of T cell clonotypes and the average total response to a non-self profile divided by the average total response to a self profile after 30,000 iterations of the update algorithm. **(E)** The proportion of non-self profiles that have a total T cell response greater than the mean response toward self profiles over time. The number of T cell clonotypes is indicated by color. **(F)** The relationship between the number of T cell clonotypes and the proportion of non-self profiles having a stronger total T cell response than the mean response to self profiles after 30,000 cycles of the update algorithm. Other model parameters for all panels are: self-response threshold *τ* = 1, growth rate *ν* = ln 2 *δt*^–1^, learning rate *η* = 0.002001 *δt*^–1^ and proportion of non-zero affinities *γ* = 0.01.

A successful T cell population needs to be able to control immune response to self but at the same time must provide broad coverage against a range of unknown non-self antigens that the individual might encounter. The mean total potential T cell response to self and non-self profiles (±standard deviation) across iterations is shown for one set of simulation parameters in Figure 3A. This shows that the response to self is well controlled at the allowed threshold *τ*. By contrast, the average response to non-self pMHC profiles becomes higher as the model shapes the repertoire. However, the non-self responses become very heterogeneous. After 30,000 iterations, the response to all self profiles is at or near the allowed threshold while the majority of non-self profiles result in more T cell binding, and therefore a larger potential T cell response (Figure 3B). However, there are also a number of non-self profiles that create a lower response than that of self profiles. These presumably represent “holes” in the repertoire coverage.

We assess the ability of the reshaped repertoire to cover the potential non-self antigen pool via the two coverage measures described earlier. We ran the update algorithm a number of times with the number of T cell clonotypes (*N*) ranging between 400 and 25,600 and number of spMHC profiles (*M*) ranging between 100 and 1,600 (only running combinations where *M* < *N*). Other parameters of the simulation are detailed in Figure 3 legend. We considered the evolution of the ratio of mean self potential response to mean non-self potential response across cycles of the algorithm (Figure 3C) and see that this increases until it is above 1 (indicating higher potential response to non-self than self profiles) for all parameter sets. The repertoire coverage, using this measure, depends on the total number of T cell clonotypes in the repertoire at the start of the algorithm (Figure 3D).

The proportion of non-self profiles that the T cell population has the potential to respond to more strongly than it does to self profiles is initially low but is increased as the update algorithm shapes the repertoire (Figure 3E). The success of the repertoire under this measure is again strongly correlated to the number of clonotypes (Figure 3F).

### 3.5 Clonotype diversity and spMHC profile cross-reactivity are preserved by the update algorithm

We have demonstrated that the model described in this study produces a TCR repertoire that respects self-response thresholds, but violates the thresholds when exposed to non-self antigen profiles. It has been observed that the TCR repertoire in an individual remains diverse (many different clonotypes are present, with cross-reactivity between clonotypes and profiles) until old-age, when a few dominant clonotypes appear (31). We explored whether our selection model can retain diversity in the repertoire or whether the multiple linear constraints favor a sparse solution with few surviving clonotypes.

**Figure 4:**
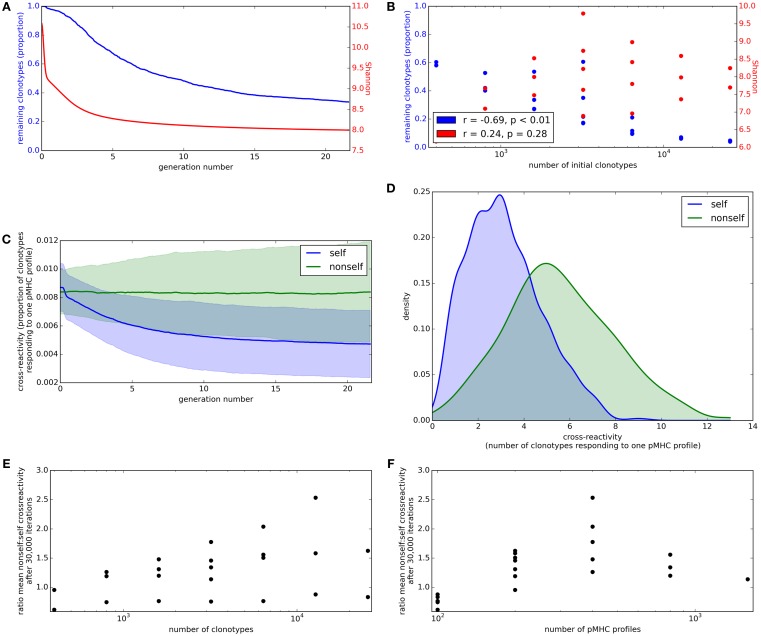
**Clonotype diversity and pMHC profile cross-reactivity are preserved by the update algorithm**. **(A)** Blue: The proportion of clonotypes (after positive selection) that are present over time during simulation of the update algorithm. Red: The Shannon entropy of the repertoire over time. Simulation implemented with *N* = 1,600 and *M* = 400. **(B)** Relationship between number of clonotypes in the simulation and proportion of clonotypes remaining (blue) or Shannon entropy of the repertoire (red) after 30,000 iterations of the update algorithm. Simulation implemented with values of *N* between 400 and 25,600 and *M* between 100 and 800, with *M* < *N*. **(C)** Cross-reactivity of T cell clonotypes against self (blue) and non-self (green) pMHC profiles over time, run with *N* = 3,200 and *M* = 400. Cross-reactivity is measured as the proportion of present clonotypes that have non-zero binding strength for a given profile. Data shown is mean cross-reactivity across all profiles ± standard deviation. **(D)** Distribution of cross-reactivity across all self (blue) and non-self (green) pMHC profiles after 30,000 iterations of the update algorithm with *N* = 3,200 and *M* = 400. Cross-reactivity is measured as the absolute number of present clonotypes that have non-zero binding strength to a profile. **(E)** Relationship between the number of clonotypes present at the start of the update algorithm and the ratio of the mean cross-reactivity against non-self profiles to the mean cross-reactivity against self profiles after 30,000 cycles of the update algorithm. **(F)** Relationship between the number of self profiles in the update algorithm and the ratio of the mean cross-reactivity against non-self profiles to the mean cross-reactivity against self profiles after 30,000 cycles of the update algorithm. Other model parameters for all panels are: self-response threshold *τ* = 1, growth rate *ν* = ln 2 *δt*^–1^, learning rate *η* = 0.002001 *δt*^–1^ and proportion of non-zero affinities *γ* = 0.01.

We first consider the proportion of starting clonotypes surviving (i.e., with an abundance greater than the lower limit defined above) as a function of time. The proportion of clonotypes present in the repertoire falls rapidly in the initial stages of repertoire reshaping and then stabilizes (Figure 4A, blue). The proportion of the initial clonotypes that remain after 30,000 cycles of the update algorithm is inversely correlated to the number of clonotypes in the simulation (Figure 4B, blue).

A key parameter of the adaptive immune system is the amount of information it can encode. The information content encoded in the repertoire (which depends on a combination of the number of different T cell clones, and also their relative size) can be captured by the Shannon Information (SI) Entropy, which is the log of the true diversity of order 1 (32). The SI coefficient of the repertoire initially decreases rapidly before stabilizing (Figure 4A, red). However, there is only a weak (and not statistically significant) correlation between the Shannon Information Entropy coefficient and the number of clonotypes in the simulation (Figure 4B, red).

Cross-reactivity, such that multiple TCRs can recognize the same pMHC profile and multiple pMHC profiles can be recognized by the same TCR, is a well-recognized feature of the T cell repertoire (4, 5). To investigate the evolution of cross-reactivity in our model, we measure the number (or proportion) of clonotypes which have non-zero binding strength for a single pMHC profile (i.e., |{*i*: *b_ij_* > 0}| for each profile *j*). The mean proportional cross-reactivity against self profiles decreases initially then begins to stabilize, while the mean cross-reactivity against non-self profiles is maintained (Figure 4C).

After running the simulation for 30,000 iterations of the update algorithm, the distributions of cross-reactivity against self and non-self profiles are clearly different (Figure 4D). The majority of non-self profiles are recognized by more TCR clonotypes than self profiles are, and the ratio of self:non-self cross-reactivity is not significantly correlated to the size of the simulation (Figures 4E,F).

### 3.6 New clonotypes can establish themselves in a stable repertoire

The TCR repertoire is constantly being updated by the introduction of new T cells from the thymus, and new clonotypes can establish themselves despite competition from the existing clonotypes. We explored whether the update algorithm of our model would allow introduction of new clonotypes. We ran the update algorithm for 30,000 iterations to produce a self tolerant and stable repertoire and then selected 10 of the clonotypes present at random. We created 10 new duplicate clonotypes, with identical spMHC profile binding strength values as the selected clonotypes, and introduced them into the repertoire at an abundance equal to the average abundance of the existing clonotypes. We then tracked both the original 10 clones and their duplicates for further iterations of the simulation.

**Figure 5:**
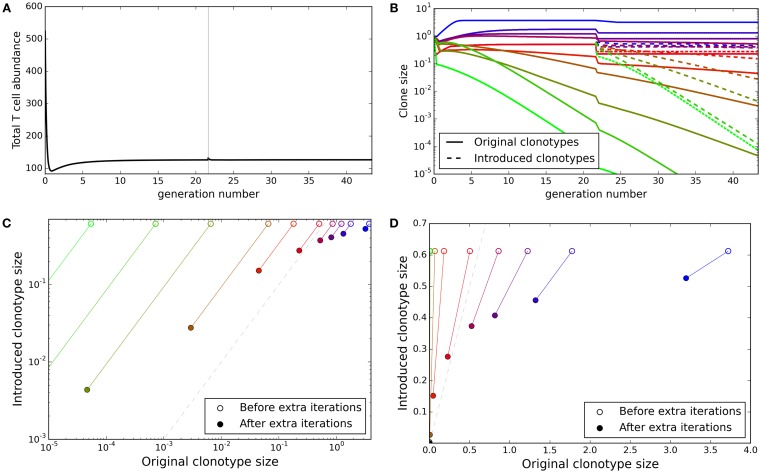
**New clonotypes can establish themselves in a stable repertoire**. **(A)** Total T cell abundance over time. Ten new clonotypes, each with self profile binding strength vector matching an existing clonotype, are introduced (at the average clonotype abundance) after 30,000 iterations of the update algorithm. **(B)** The clonal abundance of 10 selected clonotypes over time. After 30,000 iterations of the update algorithm, 10 additional clonotypes are introduced (dashed lines), each with a self profile binding strengths equal to one of the original 10 clonotypes (colors represent binding strength profiles). **(C,D)** For each of the selected original clonotypes and the binding strength-matched introduced clonotypes, the relationship between the original and match clone abundance when the new clonotype is introduced (open circles) and after running the simulation for an additional 30,000 iterations of the update algorithm (solid circles). The dashed gray line represents identical abundance of original and introduced clonotypes. Model parameters used for all panels are: self-response threshold *τ* = 1, growth rate *ν* = ln 2 *δt*^–1^, learning rate *η* = 0.002001 *δt*^–1^, proportion of non-zero affinities *γ* = 0.01, number initial clonotypes *N* = 1,000, number self profiles *M* = 100.

The total T cell abundance increases transiently as new clonotypes are introduced but quickly returns to a stable level (Figure 5A). On introduction of the new duplicate clonotypes, the abundances of the original 10 clonotypes fall in order to satisfy the self-response constraints (Figure 5B). Clonotypes with matched self-binding strength profiles are seen to tend toward the same abundance over the additional iterations of the model (Figures 5C,D). Although the abundances of the new clonotypes do not reach equality with the original clonotypes, the introduced clonotypes only disappear in cases where the original clonotypes are also deleted. The introduced clonotypes are able to remain in the repertoire even when they are introduced at a lower abundance than an already established clonotype with the same self-response profile.

## 4 Discussion

We have outlined a simple computational model by which the T cell repertoire in an individual can be continually adjusted in order to optimize the chance of a successful response to unknown pathogens while minimizing the amount of dangerous T cell response to self. From a computational perspective, the update method can be thought of as a multiplicative weight update algorithm, and is shown to rapidly converge to a solution of the constraints. From a biological perspective, the model falls within the well-established framework of APC-based self-tolerance models (see below), but introduces the key features of cross-reactivity and T cell cooperativity. The model produces the desirable features of maintaining self-reactivity within a predefined threshold, while driving the development of a diverse repertoire, which can respond effectively to a broad selection of non-self antigens. The model repertoire also reproduces the heterogeneous distribution of naive T cell clonotype abundance, which has been described by recent high throughput sequencing studies (33), and the extensive cross-reactivity which is another recently recognized feature of the T cell repertoire (5). We do not model an immune response in this work. If the APC remains in tolerogenic state, the introduction of new non-self pMHC profiles will violate the constraints, but this will result in additional T cell killing and the system will gradually readjust to remain within the immune activation threshold. If, however, the APC are switched to an immunogenic state (for example by exposure to innate immune danger signals) then crossing the threshold will result in activation of all APC bound T cells, resulting in an effector immune response.

The mechanisms whereby the vertebrate adaptive immune system avoids harmful reaction with self antigens but retains the ability to react with a large and unknown set of potential pathogens have been extensively discussed. The current molecular understanding of the stochastic recombination events, which generate adaptive immune receptors (antibody and the TCR), requires self-tolerance to be learnt rather inherited. The clonal deletion model of (1) has remained the dominant paradigm for many decades. In the context of the T cell, this paradigm posits that T cells developing in the thymus die if they react with antigens (which in the context of the thymus are assumed to be predominantly self) with an affinity above a given threshold, whose value has been estimated to correspond to a disassociation constant of approximately 6 μM (34). Indeed special molecular mechanisms exist to ensure atopic expression of a whole range of non-thymic proteins in the thymus (35), presumably to ensure robust self-tolerance. The molecular mechanism of clonal deletion has also been studied intensively (36).

More recently, a number of immunologists have proposed the need for some form of extrathymic (peripheral) tolerance, since self-reactive mature T cells have been described in many cases. Such models include those in which self/non-self discrimination was assigned to the antigen presenting cell (typically a dendritic cell) rather than the T cell (16, 17). The essence of these models was to propose that APCs exist in two different functional states. Under resting conditions (e.g., in the absence of infection), the interaction between antigen on the APC and cognate T cell induces tolerance (either deletion, or anergy). When the APC is activated (typically via the innate immune system), the same interaction leads to activation, differentiation, and T cell effector function. A fundamental feature of these models is that the APC continues to present self-antigens in both states. However, since the immune system has been “educated” to tolerize self-reactive T cells during a resting period, and the majority of antigen presenting cells at any time continue to remain in a resting state, the T cell response to self-antigens presented together with non-self by the activated antigen presenting cells is small and transitory, and does not lead to significant pathology. The model presented in this paper lies squarely within the conceptual framework of these antigen presenting cell focused models of self/non-self discrimination. However, our model simplifies the system by assuming only a single type of APC. In reality, the immune system contains a heterogeneous mixture of antigen presenting cells, with a spectrum of tolerizing or activating activity (37). The extension of our model to incorporate antigen presenting cell heterogeneity will be an important goal of future work.

The molecular mechanisms by which antigen presenting cells induce tolerance remain an open question. Tolerogenic dendritic cells, which express granzyme and perforin, and induce T cell death in an antigen specific way, have been described (15). Dendritic cells also express several members of the Tumor Necrosis Factor (TNF) family, and its cognate receptors, the TNF receptor family. Some members of this family, for example CD40 and CD40L, are known to play a critical part in T cell activation. Impairment of this interaction leads to profound immunodeficiency (38). Furthermore, CD40 expression on antigen presenting cells is modulated by T cells, and the antigen presenting cell integrates signals from multiple T cells, providing a molecular mechanism for T cell cooperativity (39). Other members of the family, which can be expressed by dendritic cells, in contrast, deliver negative signals. The most well-studied example is the Fas/FasL interaction, and impairment of this interaction leads to a breakdown of self-tolerance (40–42). TNF itself can also induce cell death via TNF receptor signals, although paradoxically it can also induce cell activation (43). The precise function of many of the more than 40 members of these families remains unknown, and their potential role in tolerance induction remains to be explored.

An interesting feature of our model is that it imposes a homeostatic limit on the total number of T cells, which depends on the self-tolerance threshold. There is extensive experimental evidence linking T cell homeostasis to inter-clonal competition for the survival/proliferation cytokine IL7 (44). An important challenge will be to integrate the phenomenon of clonal competition for a limited resource into our model. Indeed, it is possible to retain the computational infrastructure of our model but recast it emphasizing survival factors, rather than death signals. It may be the case that integration occurs in both APC and T cells, with the APC sending survival signals to bound T cells until a threshold level of binding is violated, at which point the survival signals cease. T cells would integrate the amount of survival signal received over a number of TCR–APC interactions and if this does not reach a sufficient level would die. This mechanism would increase the specificity of clonotype size adjustment, only reducing those clonotypes that repeatedly encounter APC for which the binding threshold is violated.

Of necessity, both our basic model and its implementation make a number of simplifying assumptions. The impact of some of these could be explored further by *in silico* experimentation. For example, it would be relatively straightforward to implement a model in which the proliferation of the T cells is likely to be dependent on the strength of the receptor/pMHC interaction. A more complex, but important, question to explore is the extent to which the averaging of the response over all antigen presenting cells adequately captures the real scenario, where self-tolerance must be distributed anatomically over the whole body, and where each antigen presenting cell only presents a subset of all possible self antigens.

Our model does not incorporate regulatory T cells, which are clearly an important part of the mechanisms of self-tolerance, and has been the basis for several previous theoretical models of self-tolerance (22, 23). These cells may be of particular importance for regulating those T cells with the highest affinity for self, which will still exist albeit at reduced numbers in our model, and which could be inadvertently triggered in the context of responses to non-self with potential pathogenic consequences.

The model we propose has interesting implications for inducing organ specific-tolerance in the context of allo-transplantation, which remains an unsolved problem in the context of clinical transplantation. The natural mechanisms, which maintain tolerance to self, are clearly insufficient in most cases to re-establish complete and lasting tolerance to an allograft in the absence of immune-suppression. This is perhaps not surprising since extra-thymic-tolerance is only one component of tolerance, and in isolation may be insufficient. However, with better understanding of the molecular cell biology of tolerogenic dendritic cells, it may be possible to experimentally increase the activity or number of these cells and thus re-educate the peripheral repertoire versus tolerance.

In conclusion, we propose a model of self-tolerance, which incorporates T cell cooperativity (quorum-sensing) into the mechanism for balancing self-tolerance with immuno-competence. Once a stable repertoire has been produced, we imagine that on immune challenge individual groups of antigen presenting cells are switched into an activated state, where they present antigens and drive the establishment of effector and memory cells. However, the repertoire will have learnt tolerance and hence the response to self will be small and not pathogenic. A useful feature of the model is that the threshold for self reaction can be set locally, and hence may vary in different tissues. The balance between response and tolerance may therefore be dependent on the local micro-environment. The key prediction of our model is that perturbation of either the existing T cell repertoire, or the presented pMHC landscape will cause widespread distributed changes to the overall repertoire, which will involve clones of many different specificities. The nature of these changes can be predicted by our model, and can be measured using the power of high throughput sequencing of TCR repertoires. Thus, our model will stimulate further hypothesis building and falsification, and lead to a better understanding of adaptive immunity and self-tolerance.

## Conflict of Interest Statement

The authors declare that the research was conducted in the absence of any commercial or financial relationships that could be construed as a potential conflict of interest.

## Supplementary Material

The Supplementary Material for this article can be found online at http://journal.frontiersin.org/article/10.3389/fimmu. 2015.00360
