# Supplementary material for: Immune Tolerance Maintained by Cooperative Interactions between T Cells and Antigen Presenting Cells Shapes a Diverse TCR Repertoire
Source: Front Immunol. 2015 Aug 7;6:360. doi: 10.3389/fimmu.2015.00360 (PMC4528093; doi:10.3389/fimmu.2015.00360)
Supplement: Supplementary file 1 [file Presentation_1.PDF]

Immune tolerance maintained by cooperative interactions between T cells and dendritic cells: a linear programming optimization problem.

Katharine Best [1,2], Benny Chain [1,*] and Chris Watkins [3]

[1] *Division of Infection and Immunity, University College London, UK*
[2] *CoMPLEX, University College London, UK*
[3] *Department of Computer Science, Royal Holloway, University of London, UK*

Correspondence*:
Benny Chain
Divison of Infection and Immunity, Cruciform Building, Gower Street, London, WC1E 6BT, b.chain@ucl.ac.uk

1 SUPPLEMENTARY INFORMATION

The clonotype update equation 4 in the main manuscript is an example of a type of 'multiplicative weight update algorithm' has been extensively studied in machine learning and game theory. These algorithms have excellent convergence properties; a recent expository survey of this work is **Arora et al.** (2012), which develops a unified presentation and analysis of many applications of these algorithms.

Our approach is to write the rate of change of clonotype frequencies as minus the gradient of a convex function F on vectors of N clonotype frequencies. F is defined on the positive quadrant. The updates of clonotype counts in the model then match exactly a multiplicative update algorithm for finding the minimum of a convex function. F has a unique minimum, and we can derive an estimate of the rate at which this minimum is approached.

A formal development is below: the analysis is adapted from **Arora et al.** (2012).

1.1 DERIVATION OF BOUNDS ON AVERAGE REGRET OF THE POTENTIAL FUNCTION F

We follow the approach of **Arora et al.** (2012) closely. The multiplicative weights algorithm of our figure 1 is similar to theirs, but without the weight-normalisation step. Our theorem 3 is adapted from their theorem 2.4, with the difference that in ours there is no weight normalisation, and we therefore use generalised KL-divergence, which is a measure of similarity of two positive vectors $\mathbf{q}$ and $\mathbf{x}$:

$$D(\mathbf{q}\|\mathbf{x}) = \sum_i q_i \ln\left(\frac{q_i}{x_i}\right) + \sum_i x_i - \sum_i q_i \tag{1}$$

Our final result, proposition 5 is essentially their theorem 3.11, with the difference that we take the limit in continuous time.

1.2 MODEL AND UPDATE ALGORITHM

Let there be N T-cell clones, and let the number of cells in clone i at time t be denoted x_i^t, and the vector of all N clone cell counts at time t is $\mathbf{x}^t = (x_1^t, \ldots, x_N^t)$; sometimes we will speak of clone counts without

mentioning a specific time, and denote the counts by $\mathbf{x} = (x_1, \ldots, x_N)$. Although in reality the clone counts would be positive integers, we do not consider small population size effects here, and we model the clone counts as positive real numbers.

The clone count update algorithm is as follows. Let $\mathbb{R}_+ = \{x \in \mathbb{R} \mid x > 0\}$.

Multiplicative Weights Update Algorithm

Initialisation: Fix $\eta \leq \frac{1}{2}$. Let $\mathbf{x}^1 = (x_1^1, \ldots, x_N^1) \in \mathbb{R}_+^N$.

for $t = 1, 2, \ldots, T$:

1. Let $\mathbf{m}^t = (m_1^t, \ldots, m_N^t)$ be the signals received by each of the N clonotypes; these signals are always in the range $[-1, 1]$.
2. For all i, $x_i^{t+1} = x_i^t(1 - \eta m_i^t)$

end

Figure 1. The update algorithm for clonotypes is presented here as a sequence of deterministic update steps at discrete times. At each time, the 'signal' for each clonotype is the sum of divide (negative) and die (positive) signals received by cells of that type: for the proof below, these signals are assumed in the range $[-1, 1]$, but this normalisation becomes automatic when we take the limit in continuous time.

LEMMA 1. *Let* $0 < \eta \leq \frac{1}{2}$.
For $-1 \leq m < 0$,

$$\ln \frac{1}{1 - \eta m} \leq m \ln(1 + \eta) \tag{2}$$

For $0 \leq m \leq 1$,

$$\ln \frac{1}{1 - \eta m} \leq m \ln \frac{1}{1 - \eta} \tag{3}$$

PROOF. Case $-1 \leq m < 0$: By concavity of ln, $|m| \ln(1 + \eta) \leq \ln(1 + |m|\eta)$. Remembering m is negative, it follows that:

$$\begin{aligned} m \ln(1 + \eta) &\geq -\ln(1 + |m|\eta) \\ &= \ln \frac{1}{1 - m\eta} \end{aligned}$$

Case $0 \leq m \leq 1$: Observe that $\ln \frac{1}{1-\eta}$ is convex in η, since:

$$\frac{d^2}{d\eta^2} \ln \frac{1}{1 - \eta} = \frac{1}{(1 - \eta)^2} > 0$$

Since $\ln \frac{1}{1-\eta} = 0$ when $\eta = 0$, it follows from convexity of $\ln \frac{1}{1-\eta}$ that
$\frac{1}{1-m\eta} \leq m \ln \frac{1}{1-\eta}$. ■

LEMMA 2. *For* $0 < \eta \leq \frac{1}{2}$,

$$\ln \frac{1}{1-\eta} \leq \eta + \eta^2 \tag{4}$$

and

$$\ln(1+\eta) \geq \eta - \eta^2 \tag{5}$$

PROOF. To prove (4, consider the derivatives of $u(\eta) = \ln \frac{1}{1-\eta}$ and $v(\eta) = \eta + \eta^2$:

$$\begin{aligned} u(\eta) &= \ln \frac{1}{1-\eta} & v(\eta) &= \eta + \eta^2 \\ u'(\eta) &= \frac{1}{1-\eta} & v'(\eta) &= 1 + 2\eta \\ u''(\eta) &= \frac{1}{(1-\eta)^2} & v''(\eta) &= 2 \end{aligned}$$

Observe that $u(0) = v(0)$ and $u'(0) = v'(0)$, but for small η, $u'(\eta) < v'(\eta)$, hence $u(\eta) \leq v(\eta)$ over some interval $[0, \eta^*]$, where $u(\eta^*) = v(\eta^*)$; we need to show that $\frac{1}{2} \leq \eta^*$. At $\eta = \eta^*$, the graph of $u(\eta)$ crosses that of $v(\eta)$ from below, hence $u'(\eta^*) \geq v'(\eta^*)$. Both u' and v' are monotonically increasing; observe that $u'(\eta) = v'(\eta)$ only when $\eta = 0$ or $\eta = \frac{1}{2}$, where $u'(\frac{1}{2}) = v'(\frac{1}{2}) = 2$. It follows that $\eta^* \geq \frac{1}{2}$, and $u(\eta) \leq v(\eta)$ for $\eta \in [0, \frac{1}{2}]$ as required.

For (5), consider the derivatives of $w(\eta) = \ln(1+\eta)$ and $y(\eta) = \eta - \eta^2$.

$$\begin{aligned} w(\eta) &= \ln(1+\eta) & y(\eta) &= \eta - \eta^2 \\ w'(\eta) &= \frac{1}{1+\eta} & y'(\eta) &= 1 - 2\eta \\ w''(\eta) &= \frac{-1}{(1+\eta)^2} & y''(\eta) &= -2 \end{aligned}$$

Observe that $w(0) = y(0)$, $w'(0) = y'(0)$, and $w''(\eta) > y''(\eta)$ for all $\eta > 0$. Hence $w(\eta) > y(\eta)$ for all $\eta > 0$, which includes what was to be proved. ■

THEOREM 3. *Assume that all costs* $m_i^t \in [-1, 1]$ *and* $0 < \eta \leq \frac{1}{2}$. *Then the multiplicative weights algorithm of figure 1 guarantees that after* T *rounds of learning, producing the sequence of weight vectors* $\mathbf{x}^1, \ldots, \mathbf{x}^T$, *and for* any *positive vector* $\mathbf{q}$,

$$\frac{1}{T}\sum_{t=1}^{T} \mathbf{m}^t \cdot \mathbf{x}^t \leq \frac{1}{T}\sum_{t=1}^{T} (\mathbf{m}^t + \eta|\mathbf{m}^t|) \cdot \mathbf{q} + \frac{D(\mathbf{q}\|\mathbf{x}^1)}{\eta T} \tag{6}$$

PROOF.

$$
\begin{aligned}
D(\mathbf{q}\|\mathbf{x}^{t+1}) - D(\mathbf{q}\|\mathbf{x}^{t}) &= \sum_i \left(\left(q_i \ln \frac{q_i}{x_i^{t+1}} + x_i^{t+1} - q_i \right) - \left(q_i \ln \frac{q_i}{x_i^{t}} + x_i^{t} - q_i \right) \right) \\
&= \sum_i q_i (\ln x_i^t - \ln x_i^{t+1}) + \sum_i x_i^{t+1} - x_i^t \\
&= \sum_i q_i (\ln x_i^t - \ln x_i^t (1 - \eta m_i^t)) + \sum (1 - \eta m_i^t) x_i^t - x_i^t \\
&= \sum_i q_i \ln \frac{1}{1 - \eta m_i^t} - \eta \sum_i m_i^t x_i^t \\
&= \sum_{i: m_i^t \geq 0} q_i \ln \frac{1}{1 - \eta m_i^t} + \sum_{i: m_i^t < 0} q_i \ln \frac{1}{1 - \eta m_i^t} - \eta \sum_i m_i^t x_i^t
\end{aligned}
$$

using lemma 1, we obtain

$$
\leq \ln \frac{1}{1-\eta} \sum_{i: m_i^t \geq 0} q_i m_i^t + \ln(1+\eta) \sum_{i: m_i^t < 0} q_i m_i^t - \eta \sum_i m_i^t x_i^t
$$

and using lemma 2, we obtain

$$
\begin{aligned}
&\leq (\eta + \eta^2) \sum_{i: m_i^t \geq 0} q_i m_i^t + (\eta - \eta^2) \sum_{i: m_i^t < 0} q_i m_i^t - \eta \sum_i m_i^t x_i^t \\
&= \eta \left(\sum_i (m_i^t + \eta |m_i^t|) q_i - \sum_i m_i^t x_i^t \right) \\
&= \eta \left((\mathbf{m}^t + \eta |\mathbf{m}^t|) \cdot \mathbf{q} - \mathbf{m}^t \cdot \mathbf{x}^t \right)
\end{aligned}
$$

Summing from $t = 1$ to T, we obtain:

$$
D(\mathbf{q}\|\mathbf{x}^{T+1}) - D(\mathbf{q}\|\mathbf{x}^{1}) = \eta \left(\sum_{t=1}^{T} (\mathbf{m}^t + \eta |\mathbf{m}^t|) \cdot \mathbf{q} - \mathbf{m}^t \cdot \mathbf{x}^t \right)
$$

Rearranging, we obtain:

$$
\frac{1}{T} \sum_{t=1}^{T} \mathbf{m}^t \cdot \mathbf{x}^t \leq \frac{1}{T} \sum_{t=1}^{T} (\mathbf{m}^t + \eta |\mathbf{m}^t|) \cdot \mathbf{q} + \frac{D(\mathbf{q}\|\mathbf{x}^{1}) - D(\mathbf{q}\|\mathbf{x}^{T+1})}{\eta T}
$$

KL divergence is non-negative, so that $D(\mathbf{q}\|\mathbf{x}^{T+1}) \geq 0$; we can therefore add this term to the RHS, obtaining

$$
\frac{1}{T} \sum_{t=1}^{T} \mathbf{m}^t \cdot \mathbf{x}^t \leq \frac{1}{T} \sum_{t=1}^{T} (\mathbf{m}^t + \eta |\mathbf{m}^t|) \cdot \mathbf{q} + \frac{D(\mathbf{q}\|\mathbf{x}^{1})}{\eta T}
$$

which is what was to be proved. ■

We envisage the T-cell birth-death process operating in continuous time. To approach continuous time in the limit of a sequence of small time-steps, we re-write this inequality in terms of time-steps of length δ, so that the total number of time-steps becomes $\frac{T}{\delta}$, and within each time-step there is a multiplicative update with factor $\eta\delta$:

$$\frac{1}{T/\delta}\sum_{t=1}^{T/\delta}\mathbf{m}^t\cdot\mathbf{x}^t \le \frac{1}{T/\delta}\sum_{t=1}^{T/\delta}(\mathbf{m}^t+\delta\eta|\mathbf{m}^t|)\cdot\mathbf{q}+\frac{D(\mathbf{q}\|\mathbf{x}^1)}{\eta T}$$

Using a bar to denote the time-average during the period 1 to T, and letting δ tend to zero, it follows immediately that:

COROLLARY 4.

$$\overline{\mathbf{m}\cdot\mathbf{x}} \le \overline{\mathbf{m}\cdot\mathbf{q}}+\frac{D(\mathbf{q}\|\mathbf{x}^1)}{\eta T} \tag{7}$$

assuming that the averages exist in the limit as δ tends to zero; this will be the case for the benign choices of $\mathbf{m}$ that we make below.

1.3 ON-LINE MINIMISATION OF A CONVEX FUNCTION

As **Arora et al.** (2012) describe, the multiplicative weights algorithm can be applied to on-line minimisation of a convex function, and theorem 3 can be applied to obtain explicit bounds on the average regret. Let F be a differentiable convex function on $\mathbb{R}_+^N$, and let

$$\rho = \max_{\mathbf{x},i}\left|\frac{\partial F(\mathbf{x})}{\partial x_i}\right| \tag{8}$$

That is, ρ is the maximum absolute partial gradient of F anywhere in any of the N coordinate directions. In fact it is only necessary to take ρ to be the maximum absolute such gradient that is actually encountered during the optimisation: in our optimisations, and for the F we use, ρ is unproblematically finite. Now define

$$\mathbf{m}^t = \frac{1}{\rho}\nabla F(\mathbf{x}^t) \tag{9}$$

so that $m_i^t \in [-1,1]$ for all i and t; since F is assumed convex and differentiable then for all $\mathbf{q}$ and $\mathbf{x}$:

$$F(\mathbf{x})-F(\mathbf{q}) \le \nabla F(\mathbf{x})\cdot(\mathbf{x}-\mathbf{q}) \tag{10}$$

Combining this with equation (7) we obtain

$$\overline{F(\mathbf{x})-F(\mathbf{q})} \le \overline{\nabla F(\mathbf{x})\cdot(\mathbf{x}-\mathbf{q})} \tag{11}$$

$$= \overline{\nabla F(\mathbf{x})\cdot\mathbf{x}}-\overline{\nabla F(\mathbf{x})\cdot\mathbf{q}} \tag{12}$$

$$= \rho(\overline{\mathbf{m}\cdot\mathbf{x}}-\overline{\mathbf{m}\cdot\mathbf{q}}) \tag{13}$$

$$\le \frac{D(\mathbf{q}\|\mathbf{x}^1)}{(\eta/\rho)T} \tag{14}$$

For our model, $(\eta/\rho)\frac{\partial F(\mathbf{x})}{\partial x_i}$ is the rate of growth of the count of cells in clonotype i, and we assume that is always negative for sufficiently large $\mathbf{x}$; F must therefore have an infimum in the positive quadrant

(including the axis planes, to allow for zeros): let this infimum of F be achieved at $\mathbf{q}$. Then, taking $0 \ln 0 = 0$ so that $D(\mathbf{q}||\mathbf{x}^1)$ exists even if some elements of $\mathbf{q}$ are zero, we have:

PROPOSITION 5.

$$\overline{F(\mathbf{x}) - \inf_{\mathbf{q} \in \mathbb{R}_+^N} F(\mathbf{q})} \leq \frac{D(\mathbf{q}\|\mathbf{x}^1)}{(\eta/\rho)T} \tag{15}$$

The RHS of the equation above is of the form C/T, where C is a constant, because $D(\mathbf{q}\|\mathbf{x}^1)$, η, and ρ do not vary throughout learning. From the gradient descent argument of equation 10 in the main manuscript, $F(\mathbf{x})$ decreases monotonically during learning; it follows that $F(\mathbf{x}^t)$ declines at least as fast as $\frac{1}{t^2}$. It follows that the constraints are rapidly satisfied since F rapidly approaches its minimum. However, the number N of clonotypes appears greater than the number of constraints, so that we cannot say how rapidly the clonotype concentrations $\mathbf{x}$ approach $\mathbf{q}$; this may allow clonotype diversity to persist for considerable time, even though the constraints are approximately satisfied.

REFERENCES

Arora, S., Hazan, E., and Kale, S. (2012), The Multiplicative Weights Update Method: a Meta-Algorithm and Applications., *Theory of Computing*, 8, 121–164, doi:10.4086/toc.2012.v008a006
